# The Clinical Characteristics and Serological Outcomes of Infants With Confirmed or Suspected Congenital Syphilis in Shanghai, China: A Hospital-Based Study

**DOI:** 10.3389/fped.2022.802071

**Published:** 2022-02-23

**Authors:** Yi Dai, Guanpeng Zhai, Shulian Zhang, Chao Chen, Zhihua Li, Wenjing Shi

**Affiliations:** ^1^Department of Neonatology, National Children's Medical Center, Children's Hospital of Fudan University, Shanghai, China; ^2^Department of Pediatrics, Minhang Hospital, Fudan University, Shanghai, China; ^3^Department of Pediatrics, Shanghai Sixth People's Hospital, Shanghai, China

**Keywords:** syphilis, congenital, *Treponema pallidum*, infant, newborn, syphilis serodiagnosis, prognosis

## Abstract

**Background:**

Congenital syphilis (CS) is the infection of an infant or fetus with *Treponema pallidum*. The aim of this study was to investigate the clinical features and outcomes of serology reversion in infants diagnosed with confirmed or suspected congenital syphilis (CS).

**Methods:**

Infants admitted to the neonatal department of Children's Hospital of Fudan University from 2013 to 2016 who met the case definition of CS or suspected CS were included in this study. Follow-up was performed in an outpatient clinic until reversion to non-reactivity of both toluidine red unheated serum test (TRUST) and Treponemal pallidum particle agglutination (TPPA). Follow-up data were collected until up to the end of 2019, when the last infant with CS reached 3 years of age.

**Results:**

In total, 682 infants were enrolled in this study, including 63 in the CS group and 619 in the suspected CS group. Forty-seven infants (74.6%) in the CS group had symptoms, and 57 (90.5%) had abnormal laboratory and/or long bone X-ray findings. By 6 months of age, TRUST results were negative in 53.3% of the infants with CS and in 100% of the infants with suspected CS. All the infants in the CS group returned to TRUST non-reactivity by 18 months of age. The TPPA results at 18 months of age showed that only 10.0% (3/30) of the patients in the CS group returned to non-reactivity, while a 99.6% (548/550) non-reactivity rate was observed in the suspected CS group. All the infants in the CS group returned to 19S-IgM-TPPA non-reactivity by 6 months of age.

**Conclusions:**

Although CS is an burdensome disease that may cause fetal and neonatal death, CS responds well to treatment when diagnosed and treated promptly, even when symptoms or lab/X-ray findings are present at birth.

## Introduction

Congenital syphilis (CS) is the infection of an infant or fetus with *Treponema pallidum*, and this infection is acquired during pregnancy from a mother with untreated or inadequately treated syphilis (1). CS can cause miscarriage, stillbirth, or infant death (2–4), and can also cause severe birth defects, including bone deformity, severe anemia, and nerve problems, including blindness or deafness (5). CS can be effectively prevented by universal syphilis screening at an early stage of pregnancy and treatment of those infected with penicillin (6–8), but the number of reported CS cases in China has increased nearly 25-fold, from 468 in 2000 to 12,042 in 2011. Large proportions of women with infections are not diagnosed and treated at early stages of pregnancy, and this may contribute to the increasing incidence of CS. Data from China's Information System of Prevention of Mother-to-Child Transmission of Syphilis Management showed that 79.1% of syphilis-infected women in China received antenatal care at or before 37 weeks of gestation in 2013; however, 55.4% of syphilis-infected women received no treatment or initiated treatment after 37 weeks of gestation (9). CS has become a public health problem in China, and its impact on children's health has raised widespread concern. In 2010, China issued the 2010–2020 Plan for Syphilis Control and Prevention to reach the goal of reducing the incidence of the mother-to-child transmission (MTCT) of syphilis to below 15 cases per 100,000 live births by 2020 (10). The release of the National Implementation Guidelines on preventing mother-to-child transmission of HIV, Syphilis, and Hepatitis B Programme in 2011 (11, 12), which was revised in 2015 and 2020 (13, 14), indicated the beginning of the commitment by the national government to eliminate the MTCT of CS. Between 2011 and 2018, the incidence of CS was significantly reduced from 91.6 cases per 100,000 live births to 18.4 cases per 100,000 (15).

**Figure 1:**
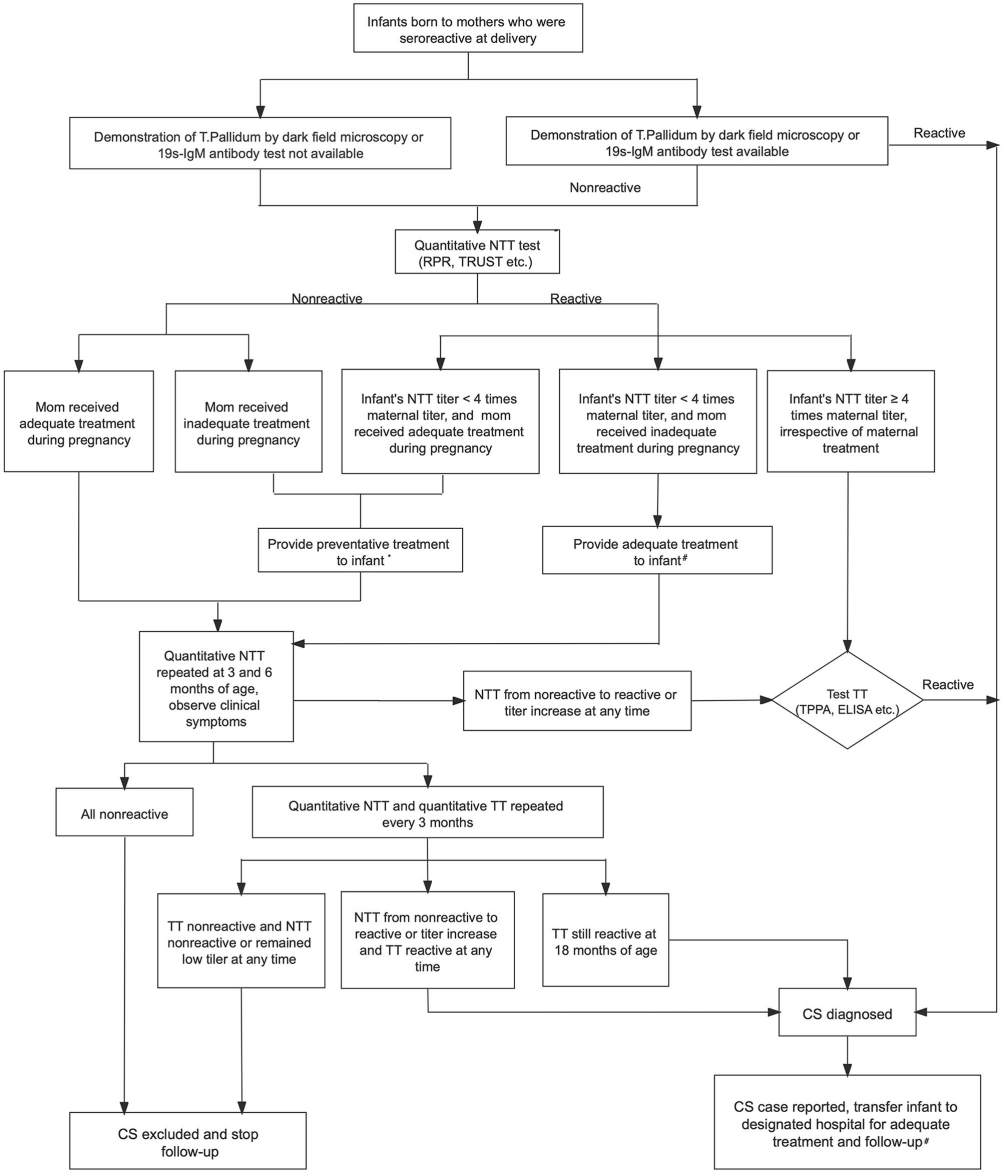
Algorithm for evaluation, treatment and follow-up of infants born to mothers with reactive serologic tests for syphilis in China (13). NTT, non-treponemal test; RPR, rapid plasma regain; TRUST, toluidine red unheated serum test; TT, treponemal test; TPPA, treponemal pallidum particle agglutination; ELISA, enzyme-linked immuno-sorbent assay; CS, congenital syphilis; CSF, cerebrospinal fluid. *Preventative treatment for patients with suspected CS: Benzathine penicillin G 50,000 U/kg IM (single dose). #Adequate treatment for patients with confirmed CS: (1) First-line therapy option: Aqueous penicillin G 50,000 U/kg IV q 12 h (≤1 wk of age), q 8 h (>1 wk), or procaine penicillin G 50,000 U/kg IM daily for 10–14 days. (2) Second-line therapy option (only if CSF is normal): Benzathine penicillin G 50,000 U/kg IM (single dose). (3) Infants should be treated as CSF abnormal if CSF examination unavailable.

The majority of infants with CS may appear normal and have no clinical or laboratory evidence of infection at birth; however, these infants may develop symptoms of disease months to years later if left untreated. The diagnosis of CS is established by the observation of spirochetes in body fluids or tissue and suggested by serologic test results. *T. pallidum* may be identified by dark field microscopy, polymerase chain reaction (PCR) testing, and fluorescent antibody or silver staining of mucocutaneous lesions, nasal discharge, vesicular fluid, amniotic fluid, placenta, umbilical cord, or tissue obtained at autopsy (16). The interpretation of reactive serological tests of the infant may be complicated by the passive transfer of maternal non-treponemal and treponemal IgG antibodies through the placenta to the fetus. Therefore, the diagnosis and management of CS is complex and requires the determination of the maternal stage of infection, adequacy of maternal treatment, and maternal response to treatment. According to Chinese guideline, all infants born to seropositive mothers require complete evaluation. Infants with confirmed CS are referred to specific health institutions for adequate treatment and long-term follow-up. Those who cannot be diagnosed of confirmed CS at birth should receive preventative treatment and are also followed up until 18 months of age when the diagnosis of CS is either confirmed or ruled out (13). Figure 1 showed the 2015 national guideline of algorithm for evaluation, treatment and follow-up of infants born to mothers with reactive serologic tests for syphilis (13).

Many studies on CS have been performed and have played an essential role in the implementation of strategies for its prevention. More recently, however, few published reports have focused on the clinical aspects of the disease. In this study, infants with confirmed or suspected CS who were admitted to Children's Hospital of Fudan University from January 2013 to December 2016 were enrolled. Serological follow-up was performed from birth as per national guidelines. The clinical characteristics and outcomes of serological reversion were compared between the confirmed CS group and the suspected CS group.

## Methods

### Patient Cohort

The study was conducted at Children's Hospital of Fudan University, a comprehensive tertiary pediatric hospital that combines medical care, teaching and research. It is located in Shanghai, one of the largest cities in China, with a total population of more than 30 million residents. Children's Hospital of Fudan University is one of the three designated medical centers for the management of CS in Shanghai and has admitted more than 95% of infants born in Shanghai to mothers with seroreactive syphilis. Infants admitted to the neonatal department of Children's Hospital of Fudan University from January 2013 to December 2016 who met the case definition of CS or suspected CS were included in the study. Data on newborn infants were collected throughout their stay in the neonatal ward. Data on infants' mothers, including antenatal screens and treatment, were collected according to the referral form from the maternal hospital. This study was approved by the Ethics Committee of the Children's Hospital of Fudan University and conducted in agreement with the ethical principles in the Declaration of Helsinki [No. 2019(241)].

Serum samples were collected immediately after admission and examined with the following tests: *Treponemal pallidum* particle agglutination (TPPA; Fujirebio Inc, Tokyo, Japan), 19S-IgM-TPPA (Euroimmun Medizinische Labordiagnostika AG, Lubeck, Germany), toluidine red unheated serum test (TRUST; Shanghai Rongsheng BioTech Co, Ltd, Shanghai, China), and TRUST titer if it was positive. For the purpose of comparison, mothers' serum samples were also collected at infant admission and examined with the TPPA and TRUST tests.

### Diagnosis and Treatment of Confirmed CS and Suspected CS

According to the national guidelines (13, 17), any neonate born to mothers who have reactive serologic test for syphilis during pregnancy as having confirmed CS if they met any one of the following laboratory criteria: (1) a positive darkfield test or PCR of lesions or body fluid; or (2) 19S-IgM-TPPA reactivity; or (3) a serum quantitative non-treponemal serologic titer that was 4-fold higher than the mother's titer. As demonstration of *Treponemal pallidum* by dark field microscopy or PCR were not available in our hospital, we defined infants as having confirmed CS if they met above criteria (2) or (3).

Infants born to mothers who had received adequate treatment but were TRUST positive or infants born to mothers who had not received treatment or had received inadequate treatment require preventative treatment with a single dose of intramuscular benzathine penicillin according to the national guidelines. Therefore, we defined these infants who are eligible to receive preventative treatment for syphilis as cases of suspected CS. We defined mothers as having received inadequate treatment if the mother had (1) treatment with a non-penicillin regimen; (2) adequate treatment but non-treponemal antibody titers increased or did not decrease at least 4-fold, or there was insufficient serological follow-up; (3) treatment administered <30 days before delivery; and (4) undocumented treatment.

Infants with non-reactive TRUST results and mothers who received adequate treatment during pregnancy were unlikely to have CS. We administered preventative treatment to these infants and provided follow-up for up to 6 months according to national guidelines, but these infants were not included in this study.

All infants born to syphilis-seropositive pregnant women were evaluated for clinical evidence (e.g., skin rash, hepatosplenomegaly, cholestatic jaundice), laboratory abnormalities [e.g., elevated liver transaminase, elevated conjugated bilirubin, anemia, thrombocytopenia, leukocytosis, elevated C reactive protein (CRP), proteinuria]. Long bone radiographs were reviewed by pediatric radiologists and determined to be consistent with CS if they demonstrated changes of osteochondritis or perichondritis (17). Cerebrospinal fluid (CSF) examination was performed for infants with confirmed CS and was considered abnormal if CSF white blood cell counts was > 25/ml, CSF protein was > 400 mg/dL and/or CSF Venereal Disease Research Laboratory (VDRL) was reactive (17).

According to national guidelines, infants with confirmed CS should receive a single dose of intramuscular benzathine penicillin if the CSF examination results are normal; otherwise, infants should receive intravenous aqueous penicillin G or procaine penicillin G intramuscular injection for 10–14 days. Infants with suspected CS should receive preventative treatment with a single dose of intramuscular benzathine penicillin. Intramuscular benzathine penicillin was not available in our hospital until 2017; therefore, both confirmed and suspected CS patients were treated with aqueous penicillin G during this study period. Infants with confirmed CS were treated for 14 days, while those with suspected CS were treated for 7 days.

### Follow-Up at the Outpatient Clinic

All the infants discharged after treatment were referred for follow-up at the neonatal clinic of Children's Hospital of Fudan University at 2, 4, 6, 9, 12, 18, and 24 months of age and then yearly until reversion to non-reactivity of both TRUST and TPPA. Follow-up data were also retrospectively collected until the end of 2019, when the last CS infant reached 3 years of age.

### Statistical Analysis

The data are presented as percentages or means (standard deviation, SD) when applicable. Independent-sample *t*-tests were used to compare the continuous parametric variables between the two groups. A continuous-calibration chi-square test was used to compare the rate between the two groups. *P* < 0.05 were considered statistically significant. Statistical analysis was conducted by using SPSS version 20.0 (SPSS Inc.).

## Results

### Characteristics of the Patients

**Figure 2:**
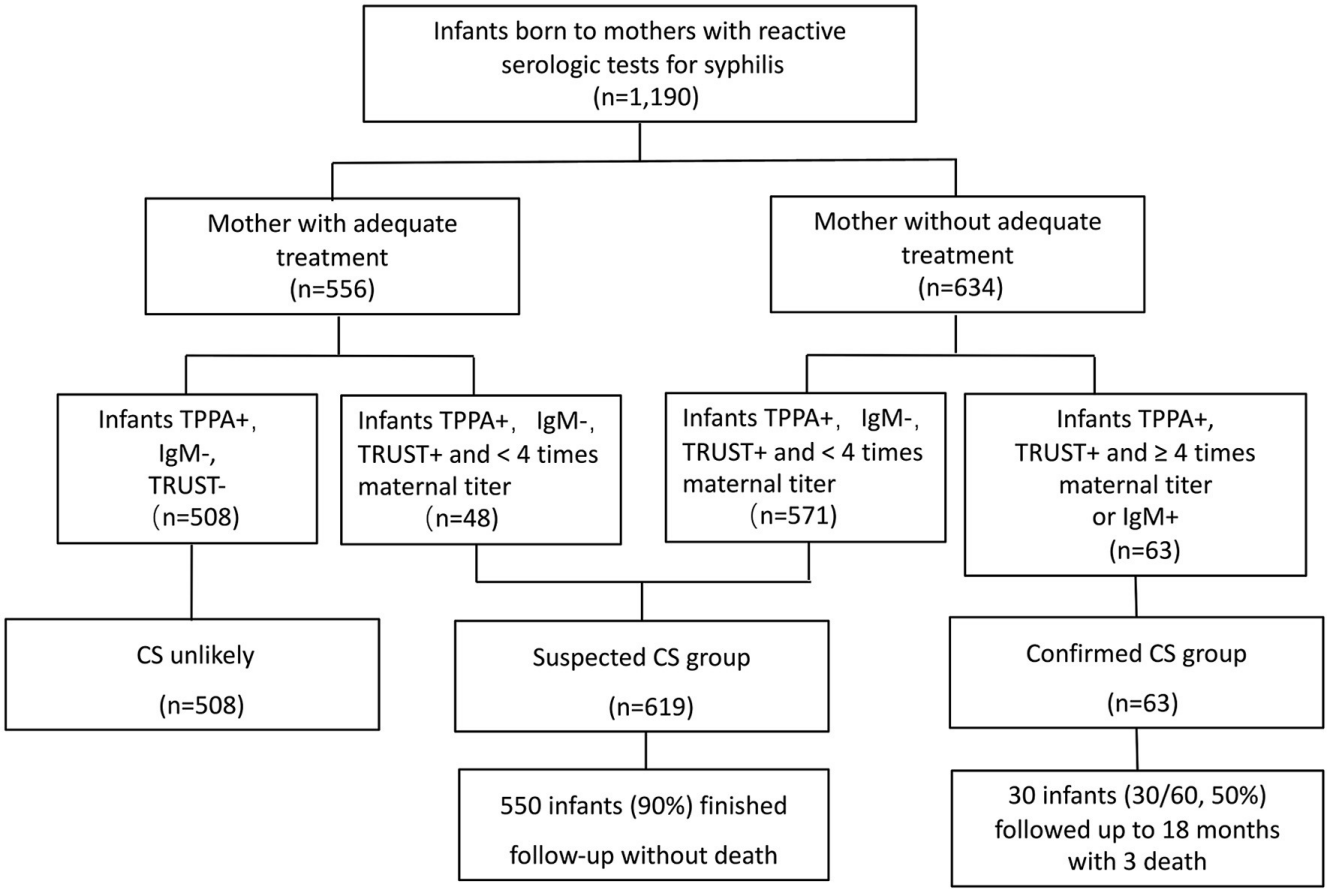
The flow chart for study groups. TPPA, treponemal pallidum particle agglutination; TRUST, toluidine red unheated serum test; CS, congenital syphilis.

Between January 2013 and December 2016, 1,190 infants born to mothers who had seropositive syphilis were admitted to the neonatal department of Children's Hospital of Fudan University for further evaluation and treatment. Among these patients, 508 infants born to mothers with adequate treatment and with non-reactive TRUST were excluded from this study. Totally 682 infants were included in this study. Of these, 63 infants met the criteria for case definition of CS (including 56 cases had positive 19S-IgM-TPPA, and seven had both positive 19S-IgM-TPPA and 4-fold higher TRUST titers than that of the mother); this group was termed the CS group. Twenty-three infants in the CS group, whose mothers were not tested for syphilis, were admitted for different reasons, including prematurity, respiratory distress, skin rash and jaundice, at a median day of life 5. The other 619 infants met the criteria for case definition of suspected CS; this group was termed the suspected CS group. The flow chart of the study infants is shown in Figure 2.

The proportion of women who received adequate treatment between the two groups was extremely low, with 7.8% infants in the suspected CS group and none in the CS group whose mothers received adequate treatment during pregnancy. 52 out of 63 (82.5%) infants in the CS group were born to mothers who did not receive any treatment. Comparison between the CS group and suspected CS group showed that there were significantly more infants with gestational age <37 weeks, the birth weights were significantly lower, and there were more infants characterized as being small for gestational age (SGA) among the infants in the CS group. There was no sex difference between the two groups. The TRUST titers of the suspected CS group were all moderate and low ( ≤ 1:16), whereas 82.5% of the CS group had high titers (≥1:32).

Basic characteristics of confirmed CS and suspected CS cases and their mothers are shown in Table 1.

**Table 1:** Basic characteristics of confirmed CS and suspected CS cases.

**Basic characteristics**	**Suspected CS group (*n* = 619)**	**CS group (*n* = 63)**	***P***
**Maternal syphilis management**
Adequate treatment, *n* (%)	48 (7.8)	0 (0)	0.042
No treatment, *n* (%)	189 (30.5)	52 (82.5)	0.000
Inadequate treatment, *n* (%)	382 (61.7)	11 (17.5)	0.000
Non-penicillin treatment, *n* (%)	129 (20.8)	2 (3.2)	
Non-treponemal test did not decreased Fourfold or increased or lack follow-up, *n* (%)	203 (32.8)	3 (4.8)	
Treatment within 30 days before delivery, *n* (%)	13 (2.1)	3 (4.8)	
Non-documented treatment, *n* (%)	37 (6.0)	3 (4.8)	
**Characteristics of infants**
Male sex, *n* (%)	335 (54.1)	38 (60.3)	0.346
GA, mean (SD), wk	39.0 (1.6)	35.6 (3.2)	0.000
GA <37 wk, *n* (%)	46 (7.4)	39 (61.9)	0.000
GA 34^0^-36^6^ wk, *n* (%)	38 (6.1)	22 (34.9)	0.000
GA 32^0^-33^6^ wk, *n* (%)	6 (1.0)	9 (14.3)	0.000
GA <32 wk, *n* (%)	2 (0.3)	8 (12.7)	0.000
BW, mean (SD), g	3,337.2 (517.7)	2,443.9 (620.0)	0.000
BW <2,500 g, *n* (%)	31 (5.0)	33 (52.4)	0.000
BW 1,500–2,499 g, *n* (%)	31 (5.0)	28 (44.4)	0.000
BW <1,500 g, *n* (%)	0 (0)	5 (7.9)	0.000
SGA, *n* (%)	28 (4.5)	12 (19.0)	0.000
TRUST non-reactive, *n* (%)	213 (34.4)	0 (0)	0.000
TRUST ≤ 1:4, *n* (%)	368 (59.5)	4 (6.3)	0.000
TRUST 1:8 or 1:16, *n* (%)	38 (6.1)	7 (11.1)	0.212
TRUST ≥ 1:32, *n* (%)	0 (0)	52 (82.5)	0.000

*CS, congenital syphilis; GA, gestational age; BW,birth weight; SGA, small for gestational age; TRUST, toluidine red unheated serum test*.

None of the infants in the suspected CS group died. After a 7-day course of aqueous penicillin treatment, 550 of 619 (90.0%) infants completed the follow-up.

In the CS group, one infant died of CS-related multiple organ failure on the first day of life, and two infants died of pneumonia in infancy after discharge. Of the other 60 infants who survived to discharge and received a 14-day course of aqueous penicillin treatment, 30 infants were lost to follow-up, and the other 30 infants were followed up for at least 18 months.

### Clinical Features in Infants Diagnosed With Confirmed CS in the Neonatal Period

Table 2 showed the clinical picture of 63 infants with confirmed CS. Among the 63 infants with CS, the percentages of prematurity, low birth weight and SGA were 61.9%, 52.4%, and 19.0%, respectively. Forty-seven infants (74.6%) in the CS group had symptoms at birth or within the first 28 days of life, and these symptoms are listed in Table 3. The most common symptoms were cholestatic jaundice and hepatomegaly, followed by splenomegaly and cutaneous lesions, all accounting for more than 50% of the total cases. Cutaneous lesions were the earliest clinical symptoms, with 22 (88.0%) of the 25 patients presenting at birth and the other 3 patients presenting at day of life 3, 7, and 25. Cutaneous lesions showed a variety of manifestations, but most patients with these lesions (21 patients, 84%) had characteristic pemphigoid lesions, others had erythema or red maculopapules, and one had eczema-like skin lesions. Symptoms with a 25–50% probability of occurrence were pneumonia, hepatitis, and gastrointestinal symptoms (including abdominal distension in 6 patients, hematochezia in 1 patient, and necrotizing enterocolitis ≥ stage 2 in 5 patients). Among the 23 patients diagnosed with pneumonia, 6 required invasive mechanical ventilation, 7 required non-invasive mechanical ventilation, 8 required nasal catheter oxygen inhalation, and 2 did not require any respiratory support. Rare symptoms include fever, petechia, nephrotic syndrome, pseudoparalysis (failure to move an extremity secondary to pain), rhinitis, vitreous opacity, and chorioretinitis, all of which occurred in fewer than 10% of the patients.

**Table 2:** The clinical pictures of 63 infants with confirmed CS.

**Patient number**	**Sex**	**BW, g**	**GA, wk**	**Admission age**	**Clinical pictures**	Long bone X-ray^*^	**CSF^#^**
1	M	1,570	31	First day	Cholestatic jaundice, hepatosplenomegaly, cutaneous lesions, GI manifestations, hepatitis	Abnormal	Normal
2	M	1,400	32	4 d	Cholestatic jaundice, pneumonitis, GI manifestations	Abnormal	CSF: WBC 47 × 10^9^ /L
3	M	3,400	38	First day	Asymptomatic	Normal	Normal
4	M	3,200	38	First day	Cholestatic jaundice, cutaneous lesions, hepatitis	Abnormal	Normal
5	F	2,275	37	First day	Asymptomatic	Abnormal	Normal
6	M	2,500	38	First day	Asymptomatic	Normal	Normal
7	F	2,990	35	12 d	Cholestatic jaundice, hepatosplenomegaly, pneumonitis, hepatitis	Abnormal	Normal
8	M	2,700	38	6 d	Cholestatic jaundice, hepatosplenomegaly, cutaneous lesions, GI manifestations, hepatitis	Abnormal	Normal
9	F	1,700	32	First day	Cholestatic jaundice, hepatosplenomegaly, cutaneous lesions, pneumonitis, fever	Abnormal	NA
10	M	1,800	36	5 d	Cholestatic jaundice, hepatosplenomegaly, cutaneous lesions	Abnormal	Normal
11	M	2,900	38	First day	Asymptomatic	Abnormal	Normal
12	F	3,000	39	9 d	Cutaneous lesions	Abnormal	Normal
13	M	1,780	33	First day	Hepatosplenomegaly, cutaneous lesions, pneumonitis, GI manifestations	NA	NA
14	M	2,000	31	First day	Cutaneous lesions, pneumonitis	NA	NA
15	F	3,080	39	First day	Cutaneous lesions	Abnormal	CSF VDRL+
16	F	1,900	35	9 d	Hepatosplenomegaly	NA	Normal
17	M	2,650	35	First day	Cholestatic jaundice, hepatosplenomegaly, cutaneous lesions, pneumonitis	Abnormal	Normal
18	F	2,220	37	First day	Cutaneous lesions	Abnormal	CSF VDRL+
19	M	2,415	33	First day	Cholestatic jaundice, hepatomegaly, pneumonitis, petechiae	Abnormal	Normal
20	F	3,050	38	First day	Asymptomatic	Abnormal	Normal
21	M	2,100	34	4 d	Cholestatic jaundice, hepatosplenomegaly, cutaneous lesions, GI manifestations, hepatitis	Abnormal	Normal
22	F	2,100	34	First day	Hepatosplenomegaly	Abnormal	Normal
23	F	1,300	27	First day	Asymptomatic	Abnormal	CSF VDRL+
24	M	3,500	38	28 d	Cholestatic jaundice, hepatitis	Abnormal	Normal
25	F	2,100	32	First day	Cutaneous lesions, GI manifestations	Abnormal	Normal
26	M	3,250	38	28 d	Cutaneous lesions, fever	Abnormal	Normal
27	F	1,545	34	First day	Cutaneous lesions	Abnormal	CSF VDRL+
28	M	2,475	39	First day	Asymptomatic	Normal	Normal
29	M	1,830	35	First day	Cholestatic jaundice, hepatosplenomegaly, cutaneous lesions, hepatitis	Abnormal	CSF VDRL+
30	M	2,500	34	First day	Cholestatic jaundice, hepatosplenomegaly, hepatitis	Abnormal	Normal
31	M	1,980	29	First day	Cholestatic jaundice, hepatosplenomegaly, pneumonitis	Abnormal	Normal
32	F	3,420	40	First day	Asymptomatic	Abnormal	Normal
33	M	2,385	36	First day	Asymptomatic	Normal	CSF VDRL+
34	F	3,000	36	First day	Cutaneous lesions, pneumonitis	Abnormal	Normal
35	M	3,250	36	First day	Asymptomatic	Normal	CSF VDRL+
36	M	3,700	40	First day	Asymptomatic	Normal	Normal
37	F	2,500	35	3 d	Hepatomegaly, GI manifestations	Normal	Normal
38	F	2,590	38	First day	Cholestatic jaundice, hepatosplenomegaly, cutaneous lesions, pneumonitis, hepatitis	Abnormal	CSF VDRL+
39	M	2,440	34	First day	Cholestatic jaundice, hepatosplenomegaly, cutaneous lesions	Abnormal	Normal
40	F	3,300	40	First day	Asymptomatic	Normal	Normal
41	M	1,900	30	10 d	Cholestatic jaundice, rhinitis, vitreous opacity	Abnormal	CSF:WBC132 × 10^9^/L, protein 2,919 mg/L
42	F	2,480	37	First day	Hepatosplenomegaly, pneumonitis, GI manifestations	NA	NA
43	M	2,700	39	First day	Asymptomatic	Abnormal	Normal
44	M	2,000	34	First day	Asymptomatic	Abnormal	Normal
45	M	2,050	31	First day	Cholestatic jaundice, hepatitis	Abnormal	CSF VDRL+
46	F	2,200	35	22 d	Cholestatic jaundice, hepatosplenomegaly, cutaneous lesions, pneumonitis, GI manifestations, hepatitis	Abnormal	CSF VDRL+
47	F	2,000	33	28 d	Cholestatic jaundice, hepatosplenomegaly, hepatitis	Abnormal	Normal
48	F	1,520	32	First day	Cholestatic jaundice, hepatosplenomegaly, cutaneous lesions, pneumonitis, GI manifestations, fever, chorioretinitis	Abnormal	NA
49	M	2,550	36	First day	Cholestatic jaundice, hepatosplenomegaly, cutaneous lesions, pneumonitis, petechiae, nephrotic syndrome	Abnormal	CSF VDRL+
50	F	2,820	36	First day	Cholestatic jaundice, hepatosplenomegaly, GI manifestations, hepatitis	Abnormal	CSF VDRL+
51	M	2,650	35	5 d	Cholestatic jaundice, pneumonitis, pseudoparalysis	Abnormal	CSF VDRL+
52	M	3,560	39	First day	Asymptomatic	Abnormal	Normal
53	M	1,440	32	27 d	Cholestatic jaundice, hepatosplenomegaly, hepatitis	Abnormal	Normal
54	F	3,500	41	First day	Asymptomatic	Normal	Normal
55	F	2,200	36	First day	Cholestatic jaundice, hepatosplenomegaly, cutaneous lesions, pneumonitis, hepatitis	Abnormal	Normal
56	M	2,430	34	First day	Cholestatic jaundice, hepatomegaly, cutaneous lesions, pneumonitis, hepatitis	Abnormal	Normal
57	F	2,460	36	First day	Asymptomatic	Abnormal	Normal
58	M	1,520	28	First day	Cholestatic jaundice, hepatosplenomegaly, pneumonitis	Abnormal	CSF VDRL+
59	M	2,800	37	First day	Pneumonitis	NA	Normal
60	M	2,085	32	First day	Hepatomegaly, pneumonitis	NA	NA
61	M	2,500	38	6 d	Cholestatic jaundice, pneumonitis, hepatitis, nephrotic syndrome	Abnormal	CSF VDRL+
62	M	1,420	30	First day	Hepatosplenomegaly, cutaneous lesions, pneumonitis, GI manifestations	Abnormal	CSF:WBC42 × 10^9^/L, VDRL+
63	M	2,800	38	First day	Cholestatic jaundice, hepatomegaly, hepatitis	Abnormal	Normal

*BW, birth weight; GA, gestational age; CSF, cerebrospinal fluid; GI, gastrointestinal; NA, non-applicable; VDRL, venereal disease research laboratory; WBC, white blood cell; F, female; M, male*.

* *Among the 63 patients, 57 received long bone X-ray. Patient number 13, 14, 16, 42, 59 refused long bone X-ray. Patient number 60 was too severe to complete the long bone X-ray and died on the first day of life*.

# *Among the 63 patients, 57 received CSF examination. Patient number 9, 13, 14, 42, 48 refused CSF examination. Patient number 60 was too severe to complete the CSF test and died on the first day of life*.

**Table 3:** Symptoms and abnormal lab or long bone X-ray presented at neonatal period in 63 infants with confirmed CS.

**Symptom**	*n* = 47 (%^*^)	**Abnormal lab or long bone X-ray**	***n* = 63 (%^#^)**
Cholestatic jaundice	30 (63.8)	Abnormal long bone X-ray^§^	48 (84.2)
Hepatomegaly	30 (63.8)	Hematologic abnormalities	44 (69.8)
Splenomegaly	25 (53.2)	Thrombocytopenia	27 (42.9)
Cutaneous lesions	25 (53.2)	Anemia	20 (31.7)
Pneumonitis	23 (48.9)	Leukocytosis	39 (61.9)
Hepatitis	18 (38.3)	Leukopenia	1 (1.6)
Gastrointestinal manifestations	12 (25.5)	Elevated CRP	32 (50.8)
Fever	3 (6.4)	Abnormal CSF	18 (31.6)
Petechiae	2 (4.3)	Reactive VDRL	16 (28.1)
Nephrotic syndrome	2 (4.3)	Elevated WBC count or protein	3 (5.3)
Pseudoparalysis	1 (2.1)	Proteinuria	4 (6.3)
Rhinitis	1 (2.1)		
Vitreous opacity	1 (2.1)		
Chorioretinitis	1 (2.1)		

*CRP, C reactive protein; CSF, cerebrospinal fluid; VDRL, venereal disease research laboratory; WBC, white blood cell*.

* *Percentages calculated on the 47 symptomatic patients. Numbers do not reflect the frequency of symptoms among all infants with CS*.

# *Percentages calculated on the 63 patients with CS*.

§ *Among the 63 children, 57 received long bone X-ray and CSF examination. Parents of the other 5 patients refused to be examined; One death was too severe to complete the long bone X-ray and CSF test. Percentages calculated on the 57 patients with long bone X-ray and CSF results. Abnormal X-ray was defined as osteochondritis or perichondritis of long bone*.

*Thrombocytopenia: platelet count <150 × 10^9^ /L; Anemia: hemoglobin <145 g/L; Leukocytosis: WBC count >10 × 10^9^ /L; Leukopenia: WBC count <4 × 10^9^ /L; Elevated CRP: CRP > 8 mg/L; Abnormal CSF: leukocytes > 25/ml or protein > 400 mg/L in cerebrospinal fluid or VDRL reactive. Proteinuria: urine protein > 30 mg/dl for urinalysis*.

Abnormal laboratory and/or X-ray findings were identified in 57 out of 63 (90.5%) patients in the CS group. Long bone abnormalities, including lucent bands under the provisional calcification zone of the long bone metaphysis (*n* = 9), irregular metaphysis with serrated appearance (*n* = 37), and periosteal reaction (*n* = 2), occurred in 48 patients. There was one patient with severe metaphyseal osseous destruction accompanied by pathological fracture of the distal left femur. The infant showed signs of pseudoparalysis. Hematological abnormalities were detected in 44 of the 63 infants (69.8%); among them, 39 infants had leukocytosis with a median white blood cell (WBC) of 26.3 × 10^9^ cells/L (range 12.11 ~ 56.3 × 10^9^ cells/L), one had leukopenia with WBC count of 3.5 × 10^9^ cells/L, 27 had thrombocytopenia with a median platelet count of 66 × 10^9^ cells/L (range 27 ~ 138 × 10^9^ cells/L) and 20 had anemia with a median hemoglobin level of 118.5 g/L (range 80 ~ 137 g/L). CRP was increased in 32 of 63 infants with a median value of 85.5 mg/L (range 15 ~ maximum, normal range <8 mg/L, maximum 160 mg/L). CSF abnormalities were observed in 31.6% (18/57) of infants, and proteinuria was detected in 4 infants. Abnormal lab or long bone X-ray present at neonatal period in 63 infants with confirmed CS are also described in Table 3.

### Serology Follow-Up of Two Groups

**Figure 3:**
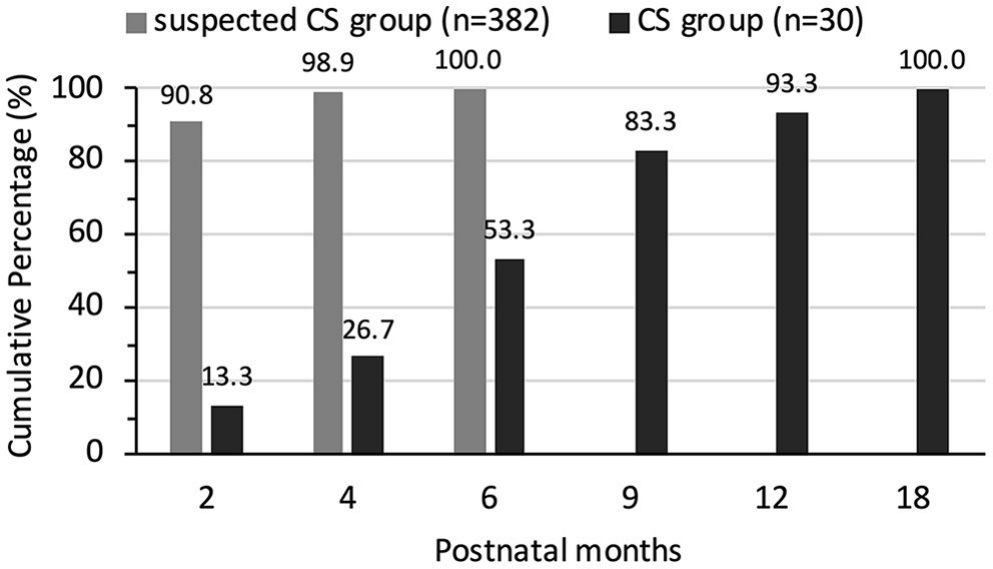
The cumulative percentage of reversion to TRUST non-reactivity in suspected CS group and CS group.

Serum TRUST result were positive at birth in 100.0% (63/63) of the infants in the CS group and 65.6% (406/619) of the infants in the suspected CS group. There were no cases of serum TRUST results changing from negative to positive or the titer increasing during follow-up. Posttreatment TRUST reversion to non-reactivity was documented in 30 patients in the CS group and 382 patients in the suspected CS group. Posttreatment TRUST reversion tended to occur earlier in infants without CS. By 6 months of age, TRUST results were negative in 53.3% of the infants with CS and in 100% of the infants in the suspected CS group. All the infants in the CS group returned to TRUST non-reactivity by 18 months of age. The cumulative percentage of reversion to TRUST non-reactivity in two groups is shown in Figure 3.

In the suspected CS group, 548 out of 550 (99.6%) infants showed TPPA non-reactivity before 18 months of age. These infants were then excluded from the diagnosis of CS and follow-up was discontinued. The other 2 out of 550 (0.4%) infants had positive TPPA results after 18 months of age, and both were reported as confirmed CS cases at this point according to national guidelines.

**Figure 4:**
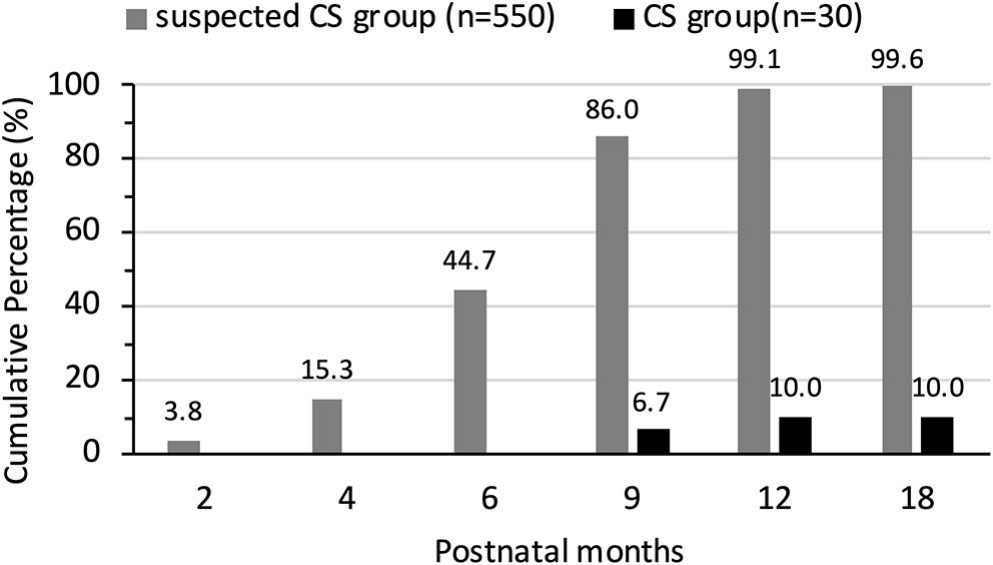
The cumulative percentage of reversion to TPPA non-reactivity in suspected CS group and CS group.

The TPPA results at 18 months of age showed that only 10.0% (3/30) of the patients in the CS group returned to non-reactivity. We were able to identify the age of reversion to non-reactivity in the other 3 infants in the CS group. TPPA non-reactivity occurred at 3, 5, and 6 years of age in these 3 infants. The cumulative percentage of reversion to TPPA non-reactivity in two groups is shown in Figure 4.

Posttreatment 19S-IgM-TPPA reversion to non-reactivity occurred at 2 and 4 months of age in 16 (53.3%) and 12 (40.0%) infants, respectively. All the infants returned to 19S-IgM-TPPA non-reactivity by 6 months of age.

## Discussion

Much has been written about CS, but in recent years, only a few studies have addressed its clinical course. This study, in addition to presenting the clinical characteristics, presents a unique comparison of serology reversion between a group of newborn infants meeting the standard case definition of CS and a group of infants with suspected CS.

Definitive diagnosis of CS is made when spirochetes are identified by darkfield microscopy, fluorescent antibody, or other specific stains in specimens from body fluids or tissue, however, most clinical settings lack the capacity to perform direct detection (18). A survey in Latin America and the Caribbean found that only two of 69 national reference and large clinical laboratories facilities still performed darkfield or direct fluorescent antibody staining for *T. pallidum* (19). PCR techniques are increasingly used; however, there is as yet no commercially available or internationally approved test for *T. pallidum* (20). Instead, most clinical laboratories utilize serological testing to infer a diagnosis of CS. Because maternal non-treponemal and treponemal IgG antibodies can be transferred from mother to infant, treponemal testing of infant serum is difficult to interpret and is not recommended (21). The finding of an infant's serum quantitative non-treponemal titer that is 4-fold higher than the maternal titer is confirmatory for CS. But the absence of such finding does not exclude the diagnosis as most infants with CS have titers that are equal to or less than the maternal titer. As anti-treponemal IgM does not cross the placenta, it is suggestive of congenital infection if detected in serum of neonatal infants. However, the use of anti-treponemal IgM is still controversial owing to limited availability of tests and inconclusive data thus far on sensitivity; their use in diagnosing CS is recommended in European (20), WHO (22), and Chinese guidelines (13), but not US Centers for Disease Control and Prevention (CDC) (21). Due to unavailability of direct detection techniques in our hospital, the confirmed CS cases were all detected by serology results. Of the 63 confirmed CS cases, 56 had positive anti-treponemal IgM, and the other 7 cases had both positive anti-treponemal IgM and 4-fold higher TRUST titers than that of the mother.

Physicians should be aware of the diverse clinical features of CS and be highly aware of CS so that a correct diagnosis can be made and treatment can be initiated early. In this study, compared to the infants who had suspected CS, infants diagnosed with confirmed CS in the neonatal period exhibited more preterm births, lower birth weights and more SGA infants. In a cross-sectional study conducted in ten maternity hospitals in Brazil, Araújo et al. found the outcome of prematurity in 15.3% of the reported cases of CS, and the non-treatment of the pregnant women or treatment with drugs other than penicillin during prenatal care (OR 3.52; 95%CI: 1.74–7.13; *p* < 0.001) were associated with higher chances of prematurity (23). In our study, more than half infants with CS were born premature, and none of the mothers received adequate treatment during pregnancy.

The 63 patients with CS had a variety of clinical manifestations, ranging from asymptomatic and without laboratory abnormalities to varying degrees of symptoms. In a serious case, the patient died of CS-related multiple organ failure in the early postnatal period, and the opportunity for treatment was lost. Similar to previous reports, the most common symptoms were cholestatic jaundice, hepatosplenomegaly, and skin lesions, all present in more than 50% of patients. Typical skin lesions, often present at birth, are frequently the indication that leads to clinical suspicion of CS. CS could also present as an atypical rash. For example, in this study, a case of an eczema-like rash commonly seen in infancy was misdiagnosed as eczema, and the diagnosis of CS was delayed until 2 weeks of life. Leung et al. reported a 2-week-old male infant with CS whose cutaneous manifestations included diffuse, erythematous keratoderma with desquamation and fissures on his hands and feet, multiple linear scaly fissures at the corners of his mouth, and onychauxis of his fingernails and toe nails, which have not been previously reported in CS (24). This suggests that for children born with rash, especially in premature babies with low birth weight or SGA, regardless of the mother's history or treatment history, the possibility of CS should be carefully considered.

Similar to previous reports, long bone abnormalities were the most common symptom and were detected in 48 out of 57 (84.2%) CS infants who had conclusive X-ray evaluation; additionally, these long bone abnormalities were most common in infants with symptomatic CS. In 6 out of 16 infants with asymptomatic CS, long bone abnormalities were the only manifestations. Although common, severe bone destructions are rare reported, and often misdiagnosed as non-accidental trauma (25–28). Only one patient in our study showed severe metaphyseal osseous destruction accompanied by fracture; this infant showed signs of pseudoparalysis. Most long bone lesions are asymptomatic and require X-ray detection. Since long bone damage is relatively specific to CS and is often used to distinguish postnatal acquired syphilis, long bone X-rays are helpful in the diagnosis of CS in patients suspected of having CS.

Hematological abnormalities, including anemia, thrombocytopenia, leukopenia, or leukocytosis, are non-specific and need to be differentiated from other causes. Tiffany Lee reported a 5-week-old male CS infant who presented with fever and a complete blood count (CBC) image suggesting malignant disease (e.g., severe leucoerythroblastic anemia with hemoglobin 1.9 g/dL, leukocytosis with WBC 53.7 × 10^9^ cells/L) (29). Thus, it is not surprising that CS is often misdiagnosed as congenital leukemia at first. Our study also found that a significant proportion of infants with CS had elevated CRP levels, with a median value of 85.5 mg/L, which was rarely reported in other studies. This suggests that in cases of unexplained CRP elevation, where there is no evidence of bacterial infection, the possibility of CS should be carefully considered.

Congenital neurosyphilis often results in significant neurologic morbidity in infants and children. Early identification and implementation of treatment are important in improving developmental outcomes and quality of life (30). In our study, CSF abnormalities, including reactive VDRL or elevated WBC count or protein levels, were detected in 31.6% (18/57) of infants with CS, while none of these infants had neurological symptoms. The diagnosis of congenital neurosyphilis is difficult to establish since the majority of infants with CS do not manifest any abnormalities on neurologic examination (31). Currently, central nervous system invasion by *T. pallidum* is usually inferred from CSF abnormalities, such as reactive VDRL, pleocytosis, and elevated protein levels (32). Although a positive VDRL in CSF is considered specific for neurosyphilis, it has limited sensitivity (33–35). Moreover, a reactive CSF VDRL in neonates may be caused by passive transfer of non-treponemal IgG antibodies from serum into the CSF. By using rabbit infectivity testing of the CSF to detect *T. pallidum* infection of the central nervous system in infants born to mothers with syphilis, Michelow et al. found that invasion of the central nervous system by *T. pallidum* occurs in 41% of infants who have clinical, laboratory, or radiographic abnormalities consistent with CS and in 60% of those who have an abnormal physical examination consistent with the diagnosis of CS; these findings indicate that central nervous system involvement is common among infants infected with syphilis (36). Therefore, once clinical, laboratory, or radiographic evaluation supports a diagnosis of congenital syphilis, therapy that is effective against central nervous system disease is warranted regardless of the results of CSF analyses (16).

In our study, the patients were divided into confirmed CS group and suspected CS group in accordance with national guideline, which were similar to the US CDC case definition of Scenario 1 (Proven or highly probable CS) and Scenario 2 (Possible CS), respectively (21). However, in terms of treatment, there are some differences between the national guideline and US CDC guideline. According to national guideline, infants with confirmed CS could receive a single dose of intramuscular benzathine penicillin if the CSF examination results are normal. While the US CDC recommended more enhanced treatment with either aqueous penicillin G or procaine penicillin G for 10 days, for infants with proven or highly probable CS, regardless of the results of CSF. In terms of infants with suspected CS, the national guidelines recommended preventative treatment with a single dose of intramuscular benzathine penicillin; while the US CDC recommended that infants with possible CS should also receive a 10 day course of aqueous penicillin G or procaine penicillin G. A single intramuscular dose of benzathine penicillin is an alternative treatment choice for infants with possible CS only if complete evaluation (i.e. CSF examination, long-bone radiographs, and CBC with platelets) was done and all normal, and follow-up is assured according to US CDC. Because successful neurosyphilis treatment requires the presence of adequate, prolonged CSF concentrations of a treponemicidal antimicrobial, benzathine penicillin should not be used to infants with neurosyphilis as it does not reliably achieve sufficient concentrations in CSF (37). Intravenous administration of aqueous penicillin G achieves adequate CSF levels and is the treatment choice for neurosyphilis. Due to the difficultly in diagnosing congenital neurosyphilis as described above, infants with confirmed CS in our study all received 14 days of aqueous penicillin G regardless of the results of CSF, which was similar to the recommendation of US CDC. Procaine penicillin G injections also achieve treponemicidal levels in CSF, but the regimen is difficult to complete because of the need for multiple intramuscular injections that can be painful to neonates. Infants in suspected CS group in this study were also treated with aqueous penicillin G, instead of one dose intramuscular benzathine penicillin as recommended by national guideline, due to the shortage of this medicine in this study period. Although benzathine penicillin is recommended as the first choice of preventing maternal-to-child transmission of syphilis and CS, the shortage of this medicine worldwide presents a major challenge in the treatment of syphilis. In a multi-country survey evaluating the shortages of benzathine penicillin for prevention of mother-to-child transmission of syphilis, 39 (41%) countries and territories reported a shortage of the medicine (38). China is not among the 39 countries with a shortage of benzathine penicillin. However, benzathine penicillin was still unavailable in many hospitals in China. A survey conducted among 948 hospitals in Shandong Province, China, reported the benzathine penicillin availability for syphilis treatment was only 45.0% in 2012, and slightly increased to 56.4% in 2018 (39). In our hospital, benzathine penicillin is unavailable until 2017, therefore, both confirmed or suspected CS cases were treated with aqueous penicillin G during the study period.

Serologic follow-up is recommended for monitoring response posttreatment as well as in suspected cases with a normal clinical examination and investigation. After adequate treatment, there was no patient with increased TRUST titers. Posttreatment TRUST reversion to non-reactivity was documented in 100% (30/30) of the patients in the CS group, although it tended to occur later than in the infants without CS. By 6 months of age, TRUST results were negative in 53.3% of the infants with CS and in 100% of infants with suspected CS. TPPA is a serum test for *Treponema pallidum*, which can passively cross the placenta. Testing for TPPA after 12 to 18 months of age has been proposed by the US CDC and national guidelines for epidemiologic surveillance purposes, as a reactive treponemal test after the disappearance of passively acquired maternal antibodies is evidence that the child had actually been infected with *T. pallidum*. In this study, after prophylactic treatment, 548 out of 550 (99.6%) infants showed TPPA non-reactivity before 18 months of age, and were then excluded from the diagnosis of CS. Only 2 (0.4%) out of 550 patients had positive TPPA results after 18 months of age, and both infants were asymptomatic during follow-up. According to national guidelines, these 2 infants were reported as confirmed CS cases at this point. In contrast, only 10% of infants with CS showed seroreversion in their treponemal tests by 18 months. In children with CS, similar to adults with syphilis, TPPA reactivity often occurs for life even after effective treatment, so it is not used as an indicator of efficacy. However, a Canadian case series of infants with CS reported that 69% of infants showed seroreversion in their treponemal test by 18 months and that infants who did not show seroreversion were more likely to have had delayed treatment (40). Early treatment is likely to alter the antibody response, making TPPA reactivity disappear over time (41).

The vagaries of maternal histories and symptoms, the lack of symptoms in newborns, and the potential consequences of delayed or missed diagnosis of CS demand a “safety first” approach to both diagnosis and treatment. On the other hand, we found that most infants with CS, when diagnosed and treated promptly, even those with symptoms or lab/X-ray findings at birth, responded well to treatment and had good outcomes. Therefore, the prognosis of children who are adequately treated for CS can be considered favorable in the absence of a very severe disease at birth and of other risk factors, especially preterm birth.

There are some limitations of our study. The main limitation was that we do not have information on infant in whom *T. pallidum* is identified by PCR, dark field microscopy, fluorescent antibody, or other specific stains in specimens from lesions to confirm CS cases, due to unavailability of these techniques in our hospital. All of our CS cases are defined by serology results, which may inevitably cause the misclassification. Secondly, infants in suspected CS group in this study were also treated with aqueous penicillin G, instead of one dose intramuscular benzathine penicillin as recommended by national guideline. Although both medicines are equally effective in the treatment of CS, aqueous penicillin G has a shorter half-life and requires a 7-day course of administration compared to a single dose of intramuscular benzathine penicillin, increasing the length of hospital stay in children with suspected CS. The third limitation was the high rate of loss to long-term follow-up in the CS group. Long-term monitoring of children with CS is difficult to implement because of low compliance by their mothers, who often lack motivation to seek health care. In our study, a high percentage of infants with CS were born to mothers who did not receive antenatal care. Lastly, the retrospective nature of this study inevitably generates inconsistencies in data collection.

## Data Availability Statement

The original contributions presented in the study are included in the article/supplementary material, further inquiries can be directed to the corresponding authors.

## Ethics Statement

The studies involving human participants were reviewed and approved by Ethics Committee of the Children's Hospital of Fudan University. Written informed consent to participate in this study was provided by the participants' legal guardian/next of kin.

## Author Contributions

YD, GZ, ZL, and WS: study design. YD, GZ, SZ, and CC: collection and analysis and interpretation of data. YD, GZ, SZ, CC, ZL, and WS: manuscript preparation and final approval. All authors contributed to the article and approved the submitted version.

## Conflict of Interest

The authors declare that the research was conducted in the absence of any commercial or financial relationships that could be construed as a potential conflict of interest.

## Publisher's Note

All claims expressed in this article are solely those of the authors and do not necessarily represent those of their affiliated organizations, or those of the publisher, the editors and the reviewers. Any product that may be evaluated in this article, or claim that may be made by its manufacturer, is not guaranteed or endorsed by the publisher.
